# Biological Activities of Lactose-Derived Prebiotics and Symbiotic with Probiotics on Gastrointestinal System

**DOI:** 10.3390/medicina54020018

**Published:** 2018-04-17

**Authors:** Arijit Nath, Gokce Haktanirlar, Áron Varga, Máté András Molnár, Krisztina Albert, Ildikó Galambos, András Koris, Gyula Vatai

**Affiliations:** 1 Department of Food Engineering, Faculty of Food Science, Szent István University, Ménesi st 44, H-1118 Budapest, Hungary; gokcehaktanirlar@gmail.com (G.H.); mr.aron.varga@gmail.com (Á.V.); molnar.a.mate@gmail.com (M.A.M.); krisztina.albert1986@gmail.com (K.A.); Koris.Andras@etk.szie.hu (A.K.); 2 Soós Ernő Water Technology Research Centre, Faculty of Engineering, University of Pannonia, Zrínyi M. u. 18, H-8800 Nagykanizsa, Hungary; galambos.i@sooswrc.hu

**Keywords:** lactose-derived prebiotics, probiotics, biological activities, gastrointestinal health

## Abstract

Lactose-derived prebiotics provide wide ranges of gastrointestinal comforts. In this review article, the probable biochemical mechanisms through which lactose-derived prebiotics offer positive gastrointestinal health are reported along with the up-to-date results of clinical investigations; this might be the first review article of its kind, to the best of our knowledge. Lactose-derived prebiotics have unique biological and functional values, and they are confirmed as ‘safe’ by the Food and Drug Administration federal agency. Medical practitioners frequently recommend them as therapeutics as a pure form or combined with dairy-based products (yoghurt, milk and infant formulas) or fruit juices. The biological activities of lactose-derived prebiotics are expressed in the presence of gut microflora, mainly probiotics (*Lactobacillus* spp. in the small intestine and *Bifidobacterium* spp. in the large intestine). Clinical investigations reveal that galacto-oligosaccharide reduces the risks of several types of diarrhea (traveler’s diarrhea, osmotic diarrhea and *Clostridium difficile* associated relapsing diarrhea). Lactulose and lactosucrose prevent inflammatory bowel diseases (Crohn’s disease and ulcerative colitis). Lactulose and lactitol reduce the risk of hepatic encephalopathy. Furthermore, lactulose, galacto-oligosaccharide and lactitol prevent constipation in individuals of all ages. It is expected that the present review article will receive great attention from medical practitioners and food technologists.

## 1. Introduction

**Figure 1:**
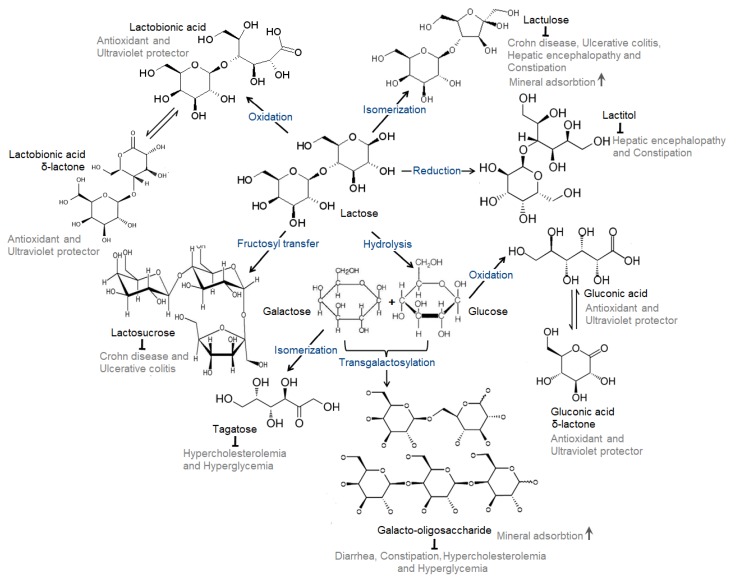
Biochemical processes for the synthesis of different types of lactose-derived prebiotics and biological outcomes due to interactions between probiotics and lactose-derived prebiotics (self-developed, concepts were adopted from Nath et al., 2016 [3], Nath et al., 2017 [7] and Gänzle et al., 2008 [13]).

In the last century, an emerging outcome of the scientific advancements in biotechnology is the synthesis of prebiotics from plant sources and dairy effluent whey, and their application as functional food supplements and therapeutics [1,2]. Lactose-derived prebiotics, such as galacto-oligosaccharide, lactulose, lactosucrose, tagatose, lactitol, lactobiono- and glucono-δ-lactone are considered a functional food supplement. Lactose-derived prebiotics are synthesized through different chemical and biochemical reactions, such as hydrolysis, transgalactosylation, isomerization, fructosyl-transfer, reduction and oxidation. In many cases, microbial fermentation processes have been adopted for lactose-based prebiotics synthesis. As the member of prebiotic family, they are resistant to gut enzymes of the upper tract, not absorbed in the gastrointestinal tract, selectively ferment, stimulate the growth and/or activity of one or limited bacteria (probiotics) in the intestine, as well as offer a number of health benefits to the consumer [3,4,5]. Lactose-derived prebiotics are of unique physico-chemical and bio-chemical importance [6,7,8] and they are confirmed as ‘safe’ by the Food and Drug Administration federal agency [9]. For these reasons, lactose-derived prebiotics are suggested for clinical purposes as therapeutics [10]. They are recommended for use in a pure form or combined with dairy-based products (yoghurt, milk and infant formulas) or fruit juices for children, young and elderly individuals [5,11,12]. The interactions between probiotics and lactose-derived prebiotics can reduce the risk of several health hazards as well as promote sustainable health [13,14]. In Figure 1, the synthesis mechanisms of different lactose-derived prebiotics through different biochemical routes and biological outcomes due to interactions between probiotics and lactose-derived prebiotics are represented.

Interactions between lactose-based prebiotics and probiotics reduce the risk of diarrhea (traveler’s diarrhea, osmotic diarrhea and *Clostridium difficile* associated relapsing diarrhea), inflammatory bowel diseases (Crohn’s disease and ulcerative colitis), constipation, colon cancer, hepatic encephalopathy (both acute encephalopathy and chronic encephalopathy), osteoporosis, hyperglycemia (Type 1 diabetes and Type 2 diabetes), hypercholesterolemia (accumulations of triglyceride-rich lipoproteins and low-density cholesterol in circulatory system), respiratory infection and allergies. Several articles (original and review articles) related to prebiotics, probiotics and their biological activities have been published by different research groups around the world. Realizing the great potential of lactose-derived prebiotics for gastrointestinal health, several clinical investigations have been performed with individuals of both genders and different ages. In this review article, the biological activities and probable mechanisms of actions of different types of lactose-derived prebiotics for gastrointestinal health are reported along with the up-to-date results of clinical inventions. 

## 2. Gut Health

It has been postulated that undernourishment leads to damage to the gut epithelium, which is the cause of a reduction of gut mediated immunity, the absorption of essential nutrients and dietary components, and loss of appetite in individuals [15]. Positive gut health signifies multiple encouraging aspects of the gastrointestinal tract, such as absence of gastrointestinal illness, effective digestion of food and absorption of nutrients, stable and substantial growth of intestinal flora, effective immune status and overall comfort in the gut (Table 1) [16]. Lactose-derived prebiotics prevent several gastrointestinal dissatisfactions and offer positive gut health [17,18,19].

### 2.1. Diarrhea 

**Figure 2:**
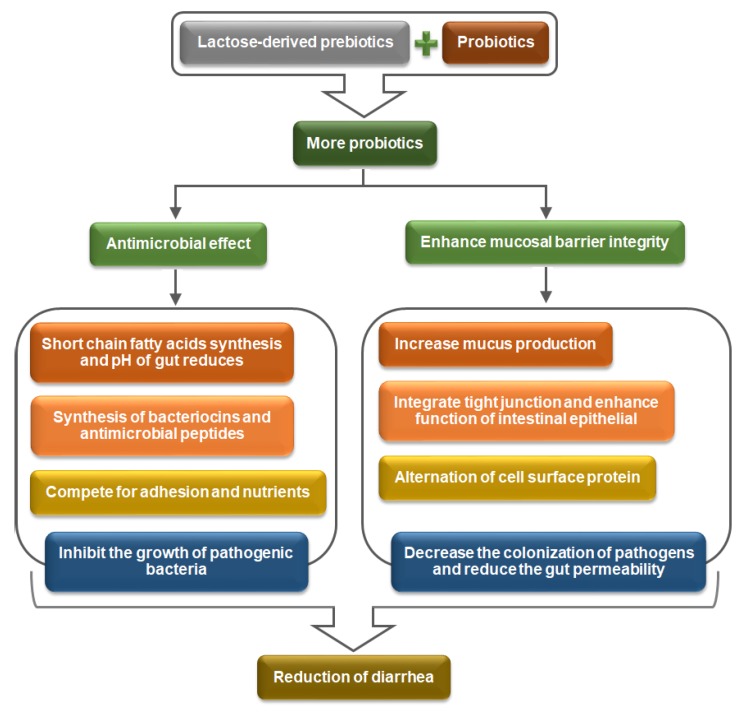
Mechanisms of diarrhea reduction by interactions between lactose-derived prebiotics and probiotics (self-developed, concepts were adopted from Saad et al., 2013 [1], Pandey et al., 2015 [14] and Fooks et al., 1999 [23]).

According to the World Health Organization, the word ‘diarrhea’ signifies three or more watery stools passing on two or more consecutive days. Diarrhea is caused by the food- or water-borne pathogens, such as *Campylobacter jejuni*, *Escherichia coli*, *Salmonella bongori*, *Shigella flexneri*, *Shigella sonnei*, *Staphylococcus aureus*, *Staphylococcal enteritis* and *Yersinia Enterocolitica* [20]. There are various mechanisms by which lactose-derived prebiotics and probiotics interactions reduce the pathogenic attack to the gastrointestinal tract and reduce the risks of diarrhea [21,22]. The major mechanisms are: (a) synthesis of antimicrobial agents (short-chain fatty acids, bacteriocins and antimicrobial peptides, mucin, collagen, fibronectin or fibrinogen, s-layer protein and lectin-like protein); and (b) improvement of the intestinal mucosal barrier defending activity through developing of a mucus layer, integrating the tight junction and the alternation of cell surface proteins. The mechanisms are reported in Figure 2 and subsequent sections.

#### 2.1.1. Biochemical Mechanisms Involved in the Reduction of Diarrhea 

##### Synthesis of Short-Chain Fatty Acids

Prebiotics are converted into lactic acid and short-chain fatty acids, such as acetic acid, propanoic acid and butyric acid due to the anaerobic fermentation by probiotics. The acidic pH prevents the colonization of a wide range of pathogens by changing the gut environment [23]. When the organic acids pass across the cell membranes of pathogens, they dissociate in the alkaline intercellular environment and acidify the cytoplasm of pathogens [24]. Diez-Gonzalez and Russell reported that organic acid dissociation in the alkaline intercellular environment of a pathogen may change the pH gradient with respect of abiotic phase. It may cause an osmotic stress to the cell, which has the fatal effect on pathogenic growth [25]. 

##### Synthesis of Bacteriocins and Antimicrobial Peptides

Probiotic synthesized bacteriocins inhibit the pathogenic population in the intestinal tract [26]. Most of the bacteriocins from lactic acid bacteria are hydrophobic or amphiphilic [27]. Bacteriocins inhibit the pathogenic population by targeting the lipid component of the cytoplasmic membrane of pathogens [28]. Class I subtype A bacteriocins are elongated, screw shaped, positively charged, amphipatic peptides, whereas class I subtype B bacteriocins are smaller globular peptides and negatively or neutral charged [29,30,31]. They form pores according to a wedge-like model in the cytoplasmic membrane of pathogens and inhibit the activities of intracellular enzymes [28]. Different types of class II bacteriocins, such as anti-listerial one-peptide pediocin-like bacteriocin, two-peptide bacteriocin, cyclic bacteriocin and linear non-pediocin-like one-peptide bacteriocin may act according to a carpet like mechanism, where bacteriocins orient parallel to the surface of the cellular membrane and interfere with the membrane or create barrel stave like pores [29]. Furthermore, probiotics induce the secretion of antimicrobial peptides (lysozyme, defensins and phospholipase) by the host, those reduce the pathogenic colonization and permeability of other antigens through the intestinal epithelial layer [32,33]. Interestingly, it has been reported that short-chain fatty acids induce the synthesis of gastrointestinal antimicrobial peptide LL-37 [34]. 

##### Competition for Adhesion 

Prebiotic-derived butyric acid enhances the growth of intestinal epithelial cells and increases the synthesis of mucin, which may reduce the bacterial adhesion on gut epithelial cells [35]. Furthermore, pathogens are unable to adhere to the intestinal surface in the presence of probiotics [36]. Probiotics are able to adhere to intestinal epithelial cells through the synthesis of surface-expressed proteins, such as collagen, fibronectin or fibrinogen [37,38,39], s-layer proteins [40,41,42,43] and lectin-like proteins [44,45]. Bernet and co-authors reported that some secreted factors are able to inhibit the binding of pathogenic bacteria to the specific receptors on the epithelium cell surface [37]. 

##### Development of the Mucus Layer 

Prebiotic-derived short-chain fatty acids promote the synthesis of intestinal tight junctional protein, i.e. mucin from goblet cells [46,47]. Furthermore, it was reported that probiotics induce the formation of a mucus layer on gut epithelial cells via the upper regulation of mucin (MUC2 and MUC3) [48,49] and their polymerization. The mucus layer acts as the first line of defense against pathogens, toxins, abrasion and dehydration [50].

##### Integrate the Tight Junction and Increase Its Barrier Activity 

Prebiotic-derived butyric acid promotes the growth of colonocyte and improves the tight-junction integrity as well as reduces gut permeability and pathogenic translocation [51,52]. Peptidoglycan in the probiotic bacterial cell membrane promotes the sealing and tightening of tight junctions via the activation of the pattern recognition receptor 2 [53,54]. Prebiotic-derived short-chain fatty acids regulate the tight junction proteins through an increase of the expression of zonula occludens 1, claudin 2, cingulin and occluding [55]. Zonula occludens proteins are responsible for developing a link between the cell cytoskeleton and the transmembrane tight junction proteins, claudins are responsible for preventing electrolyte and water losses and occludin plays a regulatory role during the integration of the tight junction [56]. Moreover, butyrate facilitates the association between claudin 1 promoter and transcription factors [57], increases adenosine monophosphate-activated protein kinase activity [52], those reduce bacterial translocation. Moreover, prebiotic-derived short-chain fatty acids integrate the gut epithelium cells via the induction of MUC2 and MUC3 synthesis from the goblet cell and develop the mucus layer on the gut epithelial cells [47,58]. However, different short-chain fatty acids are produced during prebiotic fermentation, it was reported that the butyrate has a great effect on the growth of colonocytes rather than acetate and propionate [59,60]. 

##### Alternation of Cell Surface Proteins

Probiotics have been demonstrated to induce protease-resistant immunoglobulin A synthesis and secretion through alteration of the cytokine milieu in intestinal mucosal cells. In the gut mucus layer, immunoglobulin A plays an important role in trapping pathogens or pathogenic antigens through its ability to bind with mucins (a gut epithelial surface protein) [61].

#### 2.1.2. Clinical Investigations 

Several clinical investigations have been performed to understand the effectiveness of lactose-derived prebiotics in reducing the risk of diarrhea in infants, children and elderly subjects. Maldonado et al. [62] performed a randomized, double-blind, placebo-controlled experiment with 215 infants (age 6 months) to investigate the effects of a galacto-oligosaccharide-supplemented formula (the supplementation of galacto-oligosaccharide was 4 g L^−1^, considered as control formula) and a combination of *Lactobacillus fermentum CECT5716* with similar concentration of galacto-oligosaccharide formula (considered as experimental formula) on infant diarrhea. The intervention period was six months. It was reported that at the end of experiment, the rates of incidence of gastrointestinal infection or episodes of diarrhea were 0.196 ± 0.51 incident per day and 0.363 ± 0.53 incident per day in the experimental group and control group, respectively. In another investigation, the effect of a prebiotic supplement (galacto-oligosaccharide: fructo-oligosaccharide = 9:1, concentration of prebotics mixture was 4 g L^−1^) on infant diarrhea (three or more loose or watery stools per day lasting for at least three days) was performed with 201 infants. It was reported that after twelve months of study, the rates of diarrheal episode per child at per year were 0.12 ± 0.04 and 0.29 ± 0.05 in the prebotic-supplemented group and control group, respectively. Furthermore, children with at least one episode of acute diarrhea were 10.4% and 23.8% in the prebotic supplemented group and control group, respectively [63]. Sazawal et al. [64] performed an experiment with 634 healthy children (age 1–3 years old, children did not have severe malnutrition or require hospitalization due to chronic illness) to study the effectiveness of galacto-oligosaccharide supplemented milk formula (in single sachet 32 g milk formula, considered as control formula) and milk formula containing galacto-oligosaccharides and *Bifidobacterium lactis* HN019 (galacto-oligosaccharides and *Bifidobacterium lactis* HN019 were 2.4 g per day and minimum 9.6 × 10^6^ CFU per day) on diarrhea. The intervention period was one year. It was reported that incidence of diarrhea, dysentery episodes and day of bloody diarrhea were 334, 120 and 246, respectively in experimental group, and those were 360, 150 and 283, respectively for placebo group. Another placebo-controlled, randomized, double-blind study was performed using 159 healthy adult volunteers (mean age 38 years in the experimental group and 39 years in the placebo group), who travelled for a minimum of 14 days to a country with a low or high risk of traveller’s diarrhea. Members of experimental group consumed 5.5 g of galacto-oligosachharide mixture once in a day for seven days prior to arrive at their destination and throughout their stay. On the other hand, members of placebo group received similar dose of maltodextrin for seven days. It was reported that after the treatment period, only 19 subjects in the galacto-oligosachharide treatment group (*n* = 81) and 30 subjects in the placebo group (*n* = 78) had diarrhea. Furthermore, the durations of the diarrhea were 2.368 ± 2.060 days and 4.567 ± 3.026 days in galacto-oligosachharide treatment group and placebo group, respectively [65]. 

### 2.2. Inflammatory Bowel Disease

**Figure 3:**
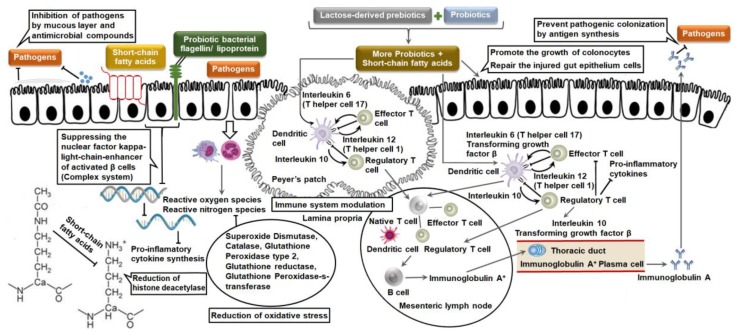
Biochemical mechanisms of the reduction of inflammatory bowel disease by interactions between lactose-derived prebiotics and probiotics (self-developed, concepts were adopted from Iacono et al., 2011 [73] and Viladomiu et al., 2013 [74]).

Inflammatory bowel diseases, such as ulcerative colitis and Crohn’s disease, are the combinations of idiopathic/abnormal intestinal conditions, characterized by a chronic relapsing course of uncontrolled inflammation in the intestine. These are autoimmune diseases, in which the body’s own immune system attacks elements of the digestive system [66]. Inflammatory bowel diseases are an abnormal mucosal immune response to antigens of pathogenic bacteria in the intestine [67] and genetic factors [68]. In Crohn’s disease, the mouth, esophagus, stomach, small intestine, large intestine and anus are affected. It is caused by attacking *Mycobacterium kansasii*, *Mycobacterium paratuberculosis*, *Listeria monocytogenes*, *Pseudomonas multophilia*, *Chlamydia trachomatis* and RNA reovirus. Crohn’s disease affected patients experience with abdominal pain and cramping, diarrhea, loss of appetite, anemia, weight loss, fever and intestinal blockage by swelling or a buildup of scar tissue in the intestinal walls. In ulcerative colitis, the colon and rectum are affected. In general, ulcerative colitis is linked with *Escherichia coli*, *Fusobacterium necrophorum*, *Streptococcus mutans*, *Shigella dysenteriae*, *Helicobacter hepaticus* and RNA viruses. The most common symptoms of ulcerative colitis are abdominal pain, cramping, diarrhea with pus, mucus or blood, nausea, loss of appetite, fatigue, anemia, weight loss and fever [69]. The etiologies of inflammatory bowel diseases are (a) disrupt the barrier function of the gastrointestinal epithelium, and the translocation of pathogens and toxins; (b) synthesis of pro-inflammatory cytokines; and (c) toxin accumulation due to oxidative stress [70]. Lactose-derived prebiotics help to reduce the risks of ulcerative colitis and Crohn’s disease via different biochemical mechanisms, including: (a) improvement of colonocytes and integration of injured gut epithelium cells; (b) down-regulation of the nuclear factor kappa-light-chain-enhancer of activated B cells; (c) reduction of oxidative stress; and (d) immunomodulation [71,72]. The mechanisms are described in Figure 3 and subsequent sections.

#### 2.2.1. Biochemical Mechanisms Involved in the Reduction of Inflammatory Bowel Disease 

##### Improvement of Colonocytes and Integration of Injured Gut Epithelium Cells 

In the intestine, lactose-derived prebiotics are broken down by probiotics and converted to different types of organic short-chain fatty acids, such as butyric acid, acetic acid, propionic acid etc., those have great importance in the metabolism. In humans, short-chain fatty acids provide about 10% of the daily caloric requirements [75]. Butyric acid acts as an energy source for the colonocyte (butyrate is an important metabolic source for the catabolism of adenosine triphosphate in host epithelial cells) [51,76] and improves the tight-junction’s integrity as well as repairs the injured gut epithelium cells, caused by the inflammatory bowel diseases. Prebiotic-derived short-chain fatty acids induce the formation of a mucus layer on gut epithelial cells [47,58], an increase of the expression of several cell membrane proteins [55] and adenosine monophosphate-activated protein kinase activity [52], those integrate the injured gut epithelium cells as well as reduce the pathogenic translocation. Consequently, the population of activated macrophages, neutrophils and synthesis of pro-inflammatory cytokines are reduced [77]. Furthermore, probiotics can interact with gut epithelial cells and reduce the water excretion, promote the secretion of anti-microbial peptides (lysozyme, defensins and phospholipase) and develop the mucus layer, those enhance the barrier function, reduce the pathogenic translocation and inhibit the synthesis of pro-inflammatory cytokines [78]. 

##### Down-Regulation of Nuclear Factor Kappa-Light-Chain-Enhancer of Activated B Cells

In intestinal epithelial cells, the nuclear factor kappa-light-chain-enhancer of activated B cells, known as nuclear factor kappa B, coordinate the immune and inflammatory responses to pathogens and other stress signals. The toll-like receptors (cell express pattern recognition receptors) in the gut epithelial layer participate to recognize the probiotic signals and capture the probiotics by binding with lectin-like proteins [79]. G-protein-coupled cell surface receptors are activated by short-chain fatty acids [80], which contribute to deactivate the expression of pro-inflammatory cytokine, such as tumor necrosis factor α and immunoregulatory cytokine interferon γ by suppressing the activity of the nuclear translocation of nuclear factor kappa B [81,82].

##### Reduction of Oxidative Stress 

Inflammation in the gut and the destruction of tight junctions are the cause of the overproduction of reactive oxygen species and reactive nitrogen species, which have a fatal effect on DNA and increase the amounts of pro-inflammatory cytokines (tumor necrosis factor α, interleukin 6 and interleukin 1β) [83,84]. The main sites for the production of reactive oxygen species and reactive nitrogen species are activated macrophages and neutrophils [85]. Short-chain fatty acids, mainly butyrate suppresses the synthesis of pro-inflammatory cytokines and pro-inflammatory mediators [77]. Butyrate also suppresses the expression of xanthine dehydrogenase and increases the synthesis of glutathione. Those reduce the purine catabolism and subsequently decrease the formation and accumulation of uric acid and reactive oxygen species [86]. Furthermore, probiotics prevent unwarranted inflammation in the gut via a reduction of oxidative stress [87,88]. The antioxidant action of probiotics can be due to their reactive oxygen species scavenging [89,90], metal ion chelation [91,92] and down-regulated ascorbate autoxidation [92] activities. Probiotic synthesized enzymes, such as superoxide dismutase [93,94], catalase [95], glutathione peroxidase type 2 [96] and peroxiredoxins [97] play a great role in reducing oxidative stress. Furthermore, probiotics reduce the oxidative stress through the synthesis of non-enzymatic antioxidants, such as folate [92], glutathione [94] and exopolysaccharide [98]. 

##### Immunomodulation 

Lactose-derived prebiotics convert to short-chain fatty acids, those enter to the cytosol of intestinal epithelial cells through a passive transport mechanism (non-ionized form) or active transport mechanism via monocarboxylate transporter 1 (Slc16a1) or the sodium-dependent monocarboxylate transporter 1 (Slc5a8) [99]. Furthermore, in immunomodulation, probiotics participate either directly with dendritic cells or indirectly via the action of M cells [100]. Subsequently, short-chain fatty acids activate differentiations and functions of dendritic cells and macrophages. Dendritic cells initiate the immune responses through the secretion of immunoregulatory cytokine, such as interleukin 10 [101] and transport to the mesenteric lymph node, where they promote the differentiation of native T cells into regulatory T cells and effector T cells. Short-chain fatty acids inhibit the maturation of the dendritic cells [102]. In immunomodulation, several mechanisms involving regulatory T cells are: (a) transfer the weak signals from antigen-presenting cells to naive/effector cells via the modulation of antigen-presenting cell activity under the involvement of regulatory T cells and co-stimulatory receptors on the antigen-presenting cell surface; (b) regulatory T cells synthesize anti-inflammatory cytokines, such as interleukin 10 and transforming the growth factor β suppress the activity of antigen-presenting cells and effector T cells; (c) regulatory T cells synthesize perforin/granzyme and induce apoptosis in effector cells (in certain situation); (d) regulatory T cells stimulate antigen-presenting cells to produce enzymes that prevent naive/effector cell proliferation and induce the expression of FoxP3 in naive cells in the presence of transforming growth factor β; (e) regulatory T cells compete with effector cells for interleukin 2 or antigen-presenting cells signals [103,104]; and (f) regulatory T cells participate with lymphocyte B to produce pathogen-specific immunoglobulin A via immunoglobulin-A^+^plasma cell formation in the presence of interleukin 10 and transforming growth factor β [105,106]. Furthermore, butyrate inhibits the growth of cancerous colonic cells by inhibiting histone deacetylase [107,108].

#### 2.2.2. Clinical Investigations 

Some clinical investigations were performed in this context. Teramoto et al. [109] performed a clinical trial with 7 patients (2 patients affected by Crohn’s disease and 5 patients affected by ulcerative colitis). All patients consumed 15 g of lactosucrose syrup (8.5 g lactosucrose in a dry basis) per day for 14 days. The authors reported that at the end of study protocol, the Bifidobacterium count was significantly increased in the feces and the population of Bacteroidaceae (pathogenic community for inflammatory bowel disease) was significantly decreased in both Crohn’s disease and ulcerative colitis patients. The relative populations of Bifidobacteria were 3.9 ± 7.2% and 12.6 ± 10.9% on the initial day and 14th day, respectively, whereas the relative populations of Bacteroidaceae were 46.9 ± 29.5% and 36.4 ± 17.0% on the initial day and 14th day, respectively. Furthermore, the authors reported that after the administration of lactosucrose, bowel movements (estimated by the frequency and regularity of defecation and the properties of feces) were improved in 1 patient with Crohn’s disease and 3 patients with ulcerative colitis. Another clinical investigation was performed by Hafer et al. [110] with 31 patients (14 patients with ulcerative colitis and 17 patients with Crohn’s disease) in the Department of Gastroenterology of the Hannover Medical School, Germany. During the investigation, 7 patients with ulcerative colitis and 8 patients with Crohn’s disease received standard medication and 15 mL of lactulose syrup (containing 10 g of lactulose) on a regular basis as adjuvant therapy and the rest of the patients, considered as the control, received standard medication according to the recommendations of the guidelines [111,112,113]. The total duration of the experiment was four months. However, there were no significant improvements in the clinical activity index [114] and Crohn’s disease activity index [115] in the ulcerative colitis and Crohn’s disease groups after the lactulose treatment, but the quality of life index [116] and inflammatory bowel disease questionnaire scores were significantly increased after the treatment with lactulose compared to the control group in the ulcerative colitis patient group.

### 2.3. Hepatic Encephalopathy 

**Figure 4:**
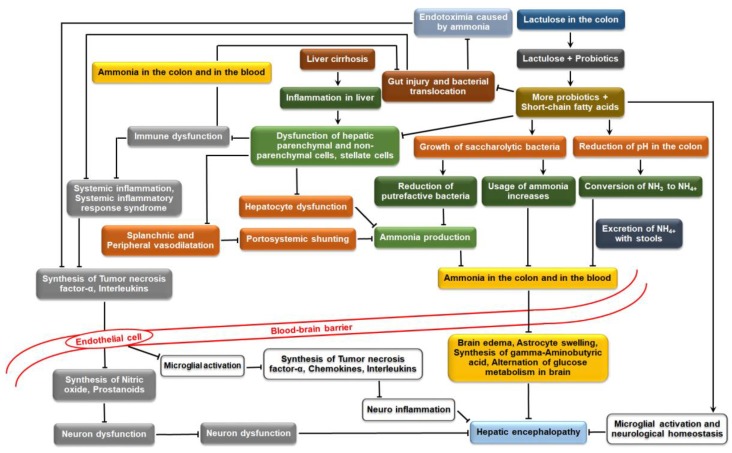
Mechanisms of the reduction of hepatic encephalopathy by interactions between lactose-derived prebiotics and probiotics (self-developed, concepts were adopted from Luo et al., 2015 [122], Nevah MI and Fallon MB, 2010 [129] and Kelly et al., 2015 [130]).

Hepatic encephalopathy represents a broad continuum of neuropsychiatric abnormalities, which is generally noticed in patients with liver dysfunction after the exclusion of other known brain diseases or patients with liver cirrhosis [117,118,119]. It can be the outcome of acute liver failure (type A hepatic encephalopathy)/portal-systemic bypass with no intrinsic hepatocellular disease (type B hepatic encephalopathy)/liver cirrhosis and portal hypertension or portal-systemic shunts (type C hepatic encephalopathy) [120]. These leads to a wide spectrum of neurological impairments, ranging from subclinical brain dysfunction to coma [121,122], and in the worst cases, it can even lead to death [117]. Hepatic encephalopathy can be scored based on the severity of the clinical manifestations, ranging from the elusive neurologic abnormalities in mild cases to coma in severe cases. Covert hepatic encephalopathy (minimal hepatic encephalopathy and West-Haven grade I hepatic encephalopathy) is considered when the patient suffers from liver cirrhosis and cognitive abnormalities without any clinical signs of brain dysfunction [123]. Overt hepatic encephalopathy (West-Haven grades II-IV hepatic encephalopathy) has a wide spectrum of clinical symptoms, including motor and neuropsychological dysfunctions [124,125]. However, hyperammonemia (the production of ammonia and its absorption in the blood) as a cause of hepatic encephalopathy has been reported in several times, inflammations (systemic inflammation and neuroinflammation) and endotoxemia were also demonstrated as the cause of hepatic encephalopathy [126]. Generally, lactulose or lactitol is prescribed for the treatment of hepatic encephalopathy [127,128]. Some biochemical mechanisms through which lactulose or lactitol reduces the risk of hepatic encephalopathy are: (a) reduction of hyperammonemia; (b) reduction of pathogenic translocation in the small intestine; (c) inhibiting pro-inflammatory interleukin synthesis; and (d) reduction of neuroinflammation. The mechanisms are reported in Figure 4 and subsequent sections. 

#### 2.3.1. Biochemical Mechanisms Involved in the Reduction of Hepatic Encephalopathy

##### Reduction of Hyperammonemia 

Lactulose or lactitol is broken down to lactic acid, short-chain fatty acids, carbon dioxide and hydrogen by probiotic bacteria in the intestinal tract. It was reported that 1 L hydrogen gas is produced from 7.0 g of lactulose [131]. Short-chain fatty acids and carbon dioxide reduce the colonic pH. Substantial hydrogen in the lower gut causes a rapid intestinal hurry, which reduce the colonic deaminating bacteria and urease positive bacteria, and consequently promotes the conversion of ammonia (NH_3_) to ammonium (NH_4+_), which is less readily absorbed by the intestinal epithelial cells. A large volume of hydrogen induces the gas-mediated activation of colonic peristalsis (rapid stool transit) and the release of ammonia through faces. Through this biochemical mechanism, ammonia absorption and the total ammonia in the portal blood are significantly decreased [131,132,133]. A similar mechanism is followed by high molecular polymeric prebiotics, such as galacto-oligosaccharides [130]. The facultative heterolactic lactobacilli and obligately homolactic lactobacilli produce several saccharolytic enzymes [134], those convert the prebiotic to short-chain fatty acids, as a main product, and to carbon dioxide, those modulate the intestinal acidity and consequently ammonium formation [131,132,133,135]. 

##### Reduction of Pathogenic Translocation in the Small Intestine 

Systemic inflammation and endotoxemia are caused by intestinal permeability, bacterial overgrowth in the small intestine and intestinal barrier dysfunction [136]. Luminal factors, such as lactulose- and lactitol-derived butyric acid, maintain the intestinal barrier (tight junction integrity) and reduce intestinal permeability, and consequently bacterial translocation. The mechanisms were described in before. As a consequence, the absorption and accumulation of bacterial toxins (lipopolysaccharides, peptidoglycan, flagellin and microbial nucleic acids) and amino acid metabolites (indoles, phenols, oxindoles and mercaptans) in the circulatory system are reduced. Endotoxemia, which activates the toll­like receptors or induces the synthesis of pro-inflammatory cytokines is also prevented [137,138]. 

##### Inhibiting Pro-Inflammatory Interleukin Synthesis 

Lactose-derived prebiotics reduce immune dysfunction (innate and adaptive immune dysfunction) through the alternation of toll-like receptors, present in parenchymal and non-parenchymal cells in the liver, and inhibiting the pro-inflammatory activities of various immune cells (secretion of pro-inflammatory interleukin 6, interleukin 18 and other mediators) on effector hepatic stellate cells [139,140]. Prebiotic-derived short-chain fatty acids reduce gut permeability and pathogenic translocation, which consequently reduces systemic inflammation as well as the synthesis of pro-inflammatory cytokines (tumor necrosis factor α, interleukin 1β) and prostanoids, those create neuroinflammation [77,126].

##### Reduction of Neuroinflammation

Probiotic and prebiotic interactions and short-chain fatty acids improve neurological health (development of brain cells, cell communication, regulation of cytokines synthesis, maintenance of neurological homoeostasis) and reduce inflammation as well as complications related with hepatic encephalopathy. Probiotics can activate the vagus nerve [141,142,143,144] as well as the central nervous system [145], affecting the immune system and influencing the synthesis of pro- and anti-inflammatory cytokines [146] that directly affect brain function. Probiotic metabolite short-chain fatty acids modulate brain development and activity by stimulating the sympathetic- and autonomic- nervous systems through G-protein-coupled receptor 41 [147], G-protein-coupled receptor 43 [148] and the blood brain barrier [149,150,151]. Short-chain fatty acids regulate microglia homoeostasis, which influences brain tissue homoeostasis as well as brain development [152,153,154], synthesis of gut peptides from enteroendocrine cells, which affect gut–brain hormonal communication [155,156,157] and the synthesis of an array of neuroactive and immunomodulatory compounds, such as γ-aminobutyric acid [158], dopamine [159], histamine [160] and acetylcholine [161]. Furthermore, short-chain fatty acids regulate the synthesis of 5-Hydroxytryptamine (serotonin) from enterochromaffin cells [162], which activates the afferent nerve to signal to the central nervous system [163]. Probiotics contribute to brain function by influencing the metabolism of tryptophan, which is an essential amino acid for serotonin synthesis. Often probiotics influence tryptophan availability through serotonergic neurotransmission [164]. Furthermore, probiotics also influence the synthesis of both neuroprotective and neurotoxic compounds through the kynurenine pathway [165].

#### 2.3.2. Clinical Investigations 

Several investigations were performed to understand the effectiveness of lactulose, lactitol and galacto-oligosaccharide for treatment of several types of hepatic encephalopathy in infants, children and elderly subjects. Watanabe et al. [166] performed a clinical investigation with 36 subclinical hepatic encephalopathy patients and 39 non-subclinical hepatic encephalopathy patients; mean age 60 ± 8 years. Patients were divided randomly and 22 patients with subclinical hepatic encephalopathy and 19 patients with non-subclinical hepatic encephalopathy were treated with an average dose of lactulose of 45 mL per day for eight weeks, and the rest of the patients did not receive lactulose. The results of the quantitative psychometric evaluation were improved at four and eight weeks after the beginning of study in subclinical hepatic encephalopathy and non-subclinical hepatic encephalopathy patients, who consumed lactulose. Another open-labeled, randomized, controlled trial was performed by Sharma et al. [167] with 140 patients, suffering from hepatic encephalopathy (cirrhosis was due to alcohol consumption, chronic hepatitis, primary biliary cirrhosis, autoimmune hepatitis and cryptogenic cirrhosis). Members of the experimental group (*n* = 70, age 48.2 ± 8.4 years) were treated with lactulose (30–60 mL of lactulose in 2–3 divided doses) with their other medication and followed up. All the patients continued treatment until they either achieved the primary end point (development of overt hepatic encephalopathy) or completed a six months follow-up period (minimum time duration) after enrollment. It was reported that 19.6% (12 patients out of 61 patients) in the lactulose treated hepatic encephalopathy group and 46.8% (30 patients out of 64 patients) in the non-lactulose treatment group developed overt hepatic encephalopathy over a fourteen-month median follow-up period (1–20 months). 

However, both lactulose and lactitol potentially reduce hepatic encephalopathy, in some cases, lactitol seems to be more efficient for the treatment of hepatic encephalopathy. A double-blind, crossover, randomized clinical trial was performed with 18 patients (mean age 54 ± 8 years) with liver cirrhosis (with spontaneous or surgically-induced shunts), and a known history and clinical signs of portal-systemic encephalopathy, to investigate the effectiveness of lactulose vs. lactitol for the treatment of portal-systemic encephalopathy. In the study protocol, two washout periods (two weeks each time) and two experimental period (two weeks each time) were considered. During the first experimental period, 10 patients were treated with 0.25 g kg^−1^ of lactose and 8 patients were treated with 0.25 g kg^−1^ of lactitol. In the second experimental period, the members of the lactitol and lactose groups were altered and the protocol followed was similar to the first experimental period. It was reported that the portal-systemic encephalopathy index was significantly decreased in the lactitol group compared to the lactulose group in the second experimental period. The portal-systemic encephalopathy indexes were 0.19 ± 0.04 and 0.27 ± 0.06 in the lactose group and lactitol group, respectively, during the first washout period; 0.25 ± 0.17 and 0.30 ± 0.17 in the lactose group and lactitol group, respectively, during first experimental period; 0.21 ± 0.08 and 0.16 ± 0.07 in the lactose group and lactitol group, respectively, during the second washout period; and 0.29 ± 0.08 and 0.16 ± 0.08 in the lactose group and lactitol group, respectively, during the second experimental period [168]. In another clinical investigation, 5 patients with cirrhosis and chronic hepatic encephalopathy (maintained stable using protein restriction and lactulose administration) were considered to study the effectiveness of lactitol vs. lactulose for the treatment of chronic hepatic encephalopathy. All patients experienced a worsening of their clinical conditions and the etiology of the cirrhosis was cryptogenic or alcoholic. Patients were hospitalized and maintained with lactulose. Initially patients were treated with lactulose for three months. After three months of monitoring with lactulose, treatment was altered to lactitol. Patients were treated with lactitol in a dose of 0.5 g per kg body weight to 0.75 g per body weight in daily for next three months. The mean values of the concentration of venous blood ammonia were 90 ± 38 µmol L^−1^, 88 ± 23 µmol L^−1^ and 75 ± 15 µmol L^−1^ after the lactulose maintenance period, lactulose treatment period and lactitol treatment period, respectively [169].

Furthermore, the reduction of hepatic encephalopathy by the symbiotic effects of lactulose and probiotics were reported by Agrawal et al. [170]. In that clinical investigation, consecutive cirrhotic patients who recovered from hepatic encephalopathy were considered. Members of the lactulose-treated group (*n* = 80) consumed 30–60 mL of lactulose in three divided doses per day, members of the probiotic treated group (*n* = 77) received three capsules of probiotics (112.5 billion viable lyophilized bacteria per capsule) per day and members of the no therapy group (*n* = 78) continued their usual food. The treatment period was twelve months. It was found that the use of lactulose and probiotics were both more effective than no therapy for secondary prophylaxis of hepatic encephalopathy treatment. Among 197 patients, 26.2% in the lactulose group, 34.4% in the probiotic group and 56.9% in the no therapy group developed an episode of overt hepatic encephalopathy over the follow-up period of twelve months.

In several cases, it was found that both lactitol and lactulose are equally effective for the treatment of hepatic encephalopathy. In a clinical investigation, the effectiveness of lactulose vs. lactitol was studied in 40 cirrhotic patients with an acute episode of portal systemic encephalopathy. Patients were randomly divided into two groups: group A (20 patients received 30 mL of lactulose (concentration 66% *w*/*v*) every 6 h) and group B (20 patients received powdered lactitol 12 g every 6 h). The duration of the treatment was five days. The results indicated that there was no significant difference in the lactulose and lactitol treatment groups. A total of 17 patients in the lactitol group and 16 patients in the lactulose group responded to therapy and a complete clinical resolution of portal systemic encephalopathy occurred in 11 patients of both groups [171]. In another comparative investigation, the effectiveness of lactulose vs. lactitol was studied in 14 patients who had liver cirrhosis (according to the biopsy report) and impairment of their psychometric performance but who were clinically stable. Patients were randomized to treatment with either lactulose, 20 mL per day or lactitol, 0.5 g per kg body weight per day for a period of two months. At the end of two months, patients followed a washout period of 2–2.5 months and subsequently they started to follow the second treatment period, similar to before. It was, however, reported that there were no significant differences in clinical status, psychometric test performance and in electroencephalogram mean cycle frequency during treatment with either lactulose or lactitol, although there were overall improvements observed in the number connection test time, digit symbol score and digit copying score with the treatment of both lactulose and lactitol [172]. Similar types of result were reported by Heredia et al. [173]. They performed a clinical investigation with 20 cirrhotic patients, mean age 54.5 ± 2.1 years with chronic or recurrent portal systemic encephalopathy. Patients consumed 15 mL of lactulose syrup (10 g of lactulose in 15 mL of solution) or 10 g lactitol powder every 6 h in daily for three months and then patients were crossed-over to the alternative treatment for the next three months, without a washout period. It was reported that the portal-systemic encephalopathy indexes [174] were 25.9 ± 11.8, 25.7 ± 9.9 and 29 ± 19.1 during entry and after treatment with lactulose and lactitol, respectively. Another clinical investigation was performed by Riggio et al. [175] with 31 liver cirrhotic patients to investigate the effectiveness of lactitol in the prevention of recurrent episodes of hepatic encephalopathy. The patients in the laltulose group (*n* = 15) received lactulose syrup 48 ± 25 mL per day and patients in the laltitol group (*n* = 14) received lactitol 36 ± 7 g per day for six months. It was reported that 9 members in the lactulose group and 10 members in the lactitol group were cured by prebiotic treatment. Morgan et al. [176] performed a randomized, double-blind, cross-over study with 9 liver cirrhosis patients. The patients consumed water solutions of sugars, lactitol (667 g L^−1^) and lactulose (445 g L^−1^) that were identical in taste, appearance and physico-chemical properties, in an initial dose of 0.75 mL per kg body weight on a regular basis for three months and subsequently crossed-over to the alternative therapy for next three months, without a washout period. After the treatment with the two prebiotics, adequate catharsis was achieved. It was found that both sugars were equally effective in the treatment of hepatic encephalopathy. Riggio et al. [177] also reported similar research findings. 

Atterbury et al. [178] reported that Neomycin-sorbitol and lactulose have a similar effect for the treatment of acute portal-systemic encephalopathy. A double-blind, randomized, clinical trial was performed with 45 patients. Subjects were divided into two categories; a neomycin-sorbitol treated group (*n* = 23), received 1.5 g of neomycin sulfate as 0.5 g tablets stat and qid thereafter and 50 mL of sorbitol syrup (60% *w*/*v*) every 1–2 h. Subsequently, the dose was reduced to 30 mL qid and adjusted to allow three bowel movements per day. The patients in the lactulose treated group (*n* = 22) received lactulose syrup (67% *w*/*v*) in a dose of 50 mL at every 1–2 h to allow two loose bowel movements per day and subsequently the dose was reduced to 30 mL qid to allow three bowel movements per day. The neomycin-sorbitol or lactulose treatment period was ten days. It was reported that 87% of patients were cured in the neomycin-sorbitol treated group and 82% of patients were cured in the lactulose treated group. Interestingly, the better effectiveness of lactulose compared with Rifaximin for the treatment of hepatic encephalopathy (the etiology of the cirrhosis was Hepatitis B virus infection or Hepatitis C virus infection or alcoholism) was reported by Paik et al. [179]. The open-label, prospective, randomized study was performed with 54 Korean patients (37 males and 17 females, mean age 55.7 year). Over seven days of treatment duration, 32 patients received rifaximin 1200 mg per day and 22 patients received lactulose 90 mL per day. It was reported that 84.4% of patients were cured by rifaximin treatment and 95.4% of patients were cured by lactulose treatment.

### 2.4. Colon Cancer 

Colorectal cancer is a malignant tumor, which appears from the inner wall of the colon or rectum. In most cases, colon cancer starts as small, noncancerous cluster of cells (adenomatous polyps) and with time, the polyps become cancer. Signs or symptoms of colon cancer include: (a) change in bowel habits (change in the consistency of stool, diarrhea and constipation for a long time); (b) feeling weak, fatigue due to losses of blood with stool and rectal bleeding and (c) abdominal discomfort (pain, cramps or gas). Generally, in the early stage, no symptoms of colon cancer appear [180,181]. Risk factors of colon cancer are: (a) age (generally it appears in older people, compare to younger people); (b) community (African-Americans have a greater risk of colon cancer compared to others); (c) a personal history of colon cancer or the presence of polyps; (d) inflammatory intestinal conditions (patients suffering from Crohn’s disease or ulcerative colitis); (e) genetic syndromes acquired from the family; (f) low fiber, high-protein and fat diets; (g) suffering from diabetes and insulin resistance; (h) obesity; (i) smoking; (j) alcoholism and (k) radiation therapy in abdomen to treat previous tumor or cancer [182,183].

**Figure 5:**
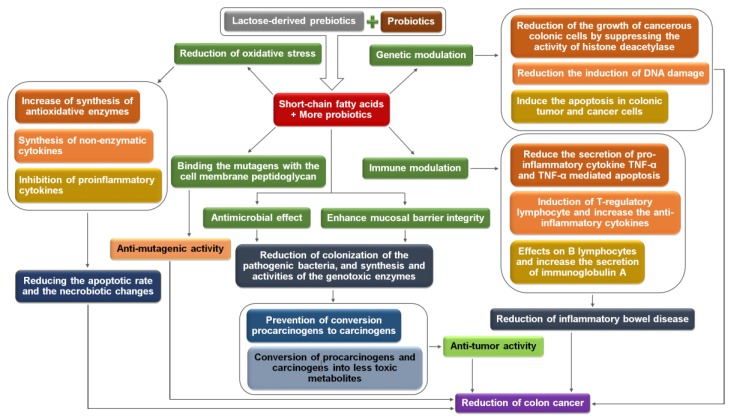
Mechanisms of the suppression of colon cancer by interactions between lactose-derived prebiotics and probiotics (self-developed, concepts were adopted from Liong, 2008 [184], Ambalam et al., 2016 [185], and Raman et al., 2013 [186]).

Interactions between lactose-derived prebiotics and probiotics reduce the risk of colon cancer [184,185]. No information about clinical investigations related to colon cancer and lactose-derived prebiotics are reported. As inflammatory bowel disease is the primary cause of colon cancer, it may be believed that lactose-derived prebiotic therapy for ulcerative colitis and Crohn’s disease patients reduces the possibility of colon cancer. The probable mechanisms related to the prevention of colon cancer due to treatment with the lactose-derived prebiotics associated with probiotics are: (a) modulation of xenobiotic enzymes; (b) immunomodulation; (c) suppression of reactive oxygen species; and (d) genetic modulation. The mechanisms are represented in Figure 5 and subsequent sections.

#### 2.4.1. Biochemical Mechanisms Involved in the Reduction of Colon Cancer 

##### Modulation of Xenobiotic Enzymes

Lactose-derived prebiotics promote the growth of probiotics, and these change the gut environment due to the formation of short-chain fatty acids, namely butyric acid, propionic acid and acetic acid [23,187]. There is a good evidence that the involvement of probiotics, and their synthesis of a range of mutagenic and genotoxic enzymes (β-glucuronidase, β-glucosidase, azoreductase and nitroreductase) and procarcinogenic compounds (indoles, phenols and amines) are the etiology of the colon cancer [188]. Prebiotic and probiotic interactions integrate the gut epithelial cells by developing and modulating the tight junctional proteins [51,55], adenosine monophosphate-activated protein kinase activity [52], mucin synthesis [47,58], antimicrobial peptides synthesis [33,34] and secretory immunoglobulin A synthesis [189]. Furthermore, probiotics produce bacteriocin [26] and eliminate the mutagenic pyrolyzates by binding with cell wall [190,191,192,193]. As a result, the colonization of pathogens in the intestine as well as the formation and activities of genotoxic enzymes and procarcinogenic compounds are significantly decreased [184,194].

##### Immunomodulation

Immunomodulation, offered by the symbiosis of lactose-derived prebiotics and probiotics, suppresses the incidence of colon cancer, which is the harsh appearance of inflammatory bowel disease and collateral tumors. The detailed mechanisms of immunomodulation due to the interactions between lactose-derived prebiotics and probiotics were explained earlier. Prebiotic and probiotic interactions influence the formation of different immune effector cells, including lymphocytes, macrophages, dendritic cells, natural killer cells, T cells and plasma cell as well as their activities (synthesis and activities of pro- and anti-inflammatory interleukins, chemokines). Transporter membrane proteins and receptors have an initial responsibility for the control and expression of the nuclear factor kappa B as well as the synthesis of pro-inflammatory cytokines [80,189]. Immunomodulatory short-chain fatty acids induce the formation of regulatory T cells, which suppress the formation and activities of effector T cells and upregulate the synthesis of anti-inflammatory interleukin 10 and transforming growth factor β, and consequently reduce the etiology of colon cancer [101]. Furthermore, probiotics influence immunomodulation via binding with toll-like receptors and subsequently deactivate the activity of nuclear factor kappa B [79]. 

##### Suppression of Reactive Oxygen Species 

Detailed mechanisms regarding the suppression of reactive oxygen species through lactose-derived prebiotics and probiotics are described in previous. Prebiotic-derived short-chain fatty acids suppress the synthesis and activities of pro-inflammatory cytokines, pro-inflammatory mediators and chemokines as well as the oxidative stress [77]. Probiotics produce several antioxidant enzymes (superoxide dismutase, catalase, glutathione peroxidase type 2 and peroxiredoxins) and non-enzymatic antioxidants (folate, glutathione, exopolysaccharide and butyrate). They reduce the formation of reactive oxygen species [88,195]. Several mechanisms, such as reactive oxygen species scavenging, metal ion chelation and reduced ascorbate autoxidation activities are responsible for the reduction of oxidative stress [87,195]. 

##### Genetic Modulation

Short-chain fatty acids are able to modify the gene-expression in tumor cells [196], reducing the induction of DNA damage [197,198], inhibiting the growth of cancerous colonic cells by suppressing the activity of histone deacetylase [107] and inducing the apoptosis in colonic tumor and cancer cells [199], which reduce the appearance of cancer in the colon.

### 2.5. Constipation 

**Figure 6:**
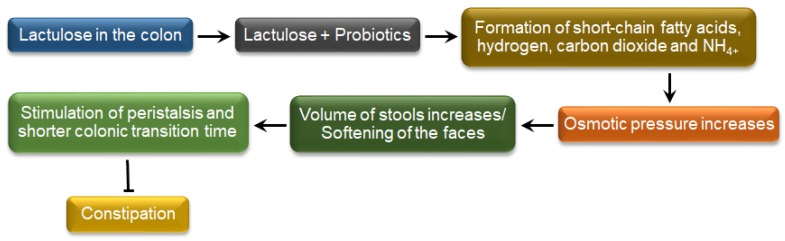
Biochemical mechanism of constipation reduction by interactions between lactose-derived prebiotics and probiotics (self-developed, concepts were adopted from Yu et al., 2017 [203], Schumann, 2002 [204] and Sahota et al., 1982 [205]).

Constipation is a common disorder in infants to elderly individuals. It refers to infrequent and/or insufficient bowel movements or hard stool, which is difficult to pass (straining). Different causes of constipation are: (a) consumption of food with lower fiber; (b) lower intake of water; (c) hormonal disorders (hypothyroidism, high levels of estrogen and progesterone); (d) stress; (e) not being active; and (f) intake of some medications, such as strong pain drugs such as narcotics, antidepressants and iron pills [200,201]. For a long time, lactose-derived prebiotics, mainly lactulose and lactitol have been popularly used for the treatment of constipation in people of different ages [202,203]. The mechanism is described in Figure 6 and the subsequent section.

#### 2.5.1. Biochemical Mechanisms Involved in the Reduction of Constipation

Prebiotic di-saccharides, such as lactulose and lactitol, are broken down by probiotics in intestine and converted to short-chain fatty acids, carbon dioxide and hydrogen. Due to the formation of lactic acid, short-chain fatty acids and carbon dioxide, the gut becomes more acidic, which leads to a reduction of ammonia absorption in the mucosa. A large volume of hydrogen, ammonia and carbon dioxide (shortly) induce the gas-mediated activation of colonic peristalsis and water to be drawn to the lower intestine, which increases the water content in the stool and makes the stool soft, increases the intestinal motility and reduces the transit time of constipated individuals [204,205]. Prebiotics with higher polymerization, such as galacto-oligosaccharide, can also considered as an osmotic laxative [206]. 

#### 2.5.2. Clinical Investigations 

Several clinical investigations have been performed to understand the effectiveness of lactulose, lactitol and galacto-oligosaccharide for the treatment of constipation in infants, children and elderly subjects. A double-blind, randomized, placebo-controlled clinical trial was performed with 84 children and adolescents (age 2–16 years) with constipation (<3 spontaneous bowel movements per week for at least twelve weeks) to assess the effectivity of lactulose, and a combination of lactulose with *Lactobacillus rhamnosus* GG for reducing constipation. The members of the experimental group (*n* = 43) received 70% of lactulose with 10^9^ colony-forming unit of *Lactobacillus rhamnosus* GG in 1 mL per kg body weight on a regular basis and members of the placebo group (*n* = 41) received lactulose with placebo food twice a day for 12 weeks. It was reported that no additional benefits were noticed in the *Lactobacillus rhamnosus* GG and lactulose treated group compared to lactulose alone. Defecation frequencies per week were 1.3 ± 1.5 and 1.6 ± 1.8 in the lactulose with *Lactobacillus rhamnosus* GG treated group and lactulose group, respectively. Spontaneous bowel movements per week were 6.1 ± 1.8 and 6.8 ± 3.1 in the lactulose with *Lactobacillus rhamnosus* GG treated group and lactulose group, respectively [207]. Contradictory results were reported by other investigators. Another randomized double-blind controlled clinical trial experiment was performed by Sadeghzadeh et al. [208] with 56 chronically constipated children (age 4–12 years). The members of the prebiotic-probiotic group received lactulose 1 mL per kg body weight per day and Protexin (mixture of probiotic *Lactobacillus acidophilus* PXN 35, *Lactobacillus casei* PXN 37, *Lactobacillus bulgaricus* PXN 39, *Lactobacillus rhamnosus* PXN 54, *Streptococcus thermophiles* PXN 66, *Bifidobacterium breve* PXN 25 and *Bifidobacterium infantis* PXN 27) one sachet daily, and the members of the placebo group received lactulose with a placebo composition. The duration of the feeding trial was four months with one week wash-out period for those who used any anticonstipation medicine. Finally, a total of 48 patients (14 males and 10 females in the intervention group, and 10 males and 14 females in the control group) finished the study protocol. It was reported that at the end of the fourth week, defecation frequencies were 0.92 ± 0.72 and 0.75 ± 0.61 in the lactulose with *Lactobacillus* group and lactulose group, respectively; stool consistencies were 0.46 ± 0.51 and 0.42 ± 0.50 in the lactulose with *Lactobacillus* group and lactulose group, respectively. Beleli et al. [209] performed an interventional, non-randomized, double-blinded, placebo-controlled, crossover study with 20 children and adolescents (age 4–16 years) to evaluate the effectiveness of galacto-oligosaccharide vs. maltodextrin for the treatment of constipation. Finally, a total of 20 subjects finished the study protocol. Members of the experimental group received a galacto-oligosaccharide solution (0.28 g mL^−1^ per day) and members of the placebo group received maltodextrin solution in a similar dose. It was reported that galacto-oligosaccharide treatment was related to an increase of bowel movement frequency, relief of defecation straining and a decreased stool consistency compared to the placebo. Sairanen et al. [210] performed a randomized, double-blind, cross-over investigation with 43 constipated elderly subjects (11 males and 32 females) with a mean age of 76 years. The study duration (total ten weeks) was divided into two-weeks baseline period, three-weeks first intervention period, two-weeks wash-out period and finally three-weeks second intervention period. The test yoghurt contained 6 g of galacto-oligosaccharide, 6 g of prunes and 3 g of linseed, whereas the placebo yoghurt was free from prunes and galacto-oligosaccharide. Both the yoghurts were prepared using *Lactobacillus acidophilus* and *Bifidobacterium lactis*. However, the subjects consumed the yoghurt two times per day (total yoghurt consumption 130 g per day) but in the first four days of the three-weeks intervention period, subjects consumed the yoghurt only one time per day. It was reported that defecation frequencies were 8.0 ± 0.6 and 7.1 ± 0.5 times per week in the test yoghurt period and control yoghurt period, respectively, whereas the defecation frequency was 5.7 ± 0.5 times per week during the baseline period. Another double-blind, two-period, cross-over study was performed with 14 constipated elderly women (mean age of 80 years) to investigate the effectivity of galacto-oligosaccharide-fortified yoghurt (9 g of galacto-oligosaccharide with 130 g of yoghurt per day) on constipation. The total study period was six weeks. The authors reported that weekly defecation frequencies were significantly increased in the galacto-oligosaccharide-fortified yoghurt treated group [206]. Van der Spoel et al. [211] performed a double-blind, placebo-controlled, randomized study with 308 patients to investigate the effectiveness of lactose on the reduction of constipation in patients who suffered from multiple organ failure and were unable to defecate, supported by mechanical ventilation and intravenous circulatory support. Patients were divided into a placebo group, lactulose treatment group and polyethylene glycol treatment group. The lactulose solution (13 g of lactulose in 100 mL of sterile water), polyethylene glycol solution (13.125 g of polyethylene glycol in 100 mL of sterile water) and 100 mL of sterile water were administrated at 8 h intervals starting at 14:00 h (afternoon) on the third day after admission. It was reported that defecation during the study period improved by 31%, 74% and 69% patients in the placebo group, polyethylene glycol group and lactulose group, respectively. The patients in the lactulose group, polyethylene glycol group and placebo group produced stools after a median of 36 h, 44 h and 75 h, respectively.

## 3. Concluding Remarks and Future Prospects

Due to irregular food habits, insufficient consumption of a healthy diet, unhealthy lifestyles and improper genetic factors; abnormal gastrointestinal symptoms are common in the community. Gastrointestinal complications include diarrhea, constipation, ulcerative colitis, Crohn’s disease, hepatic encephalopathy, colon cancer, etc. Often, lactose-derived prebiotics are consumed alone as well as with dairy products or fruit juices. The Food and Drug Administration federal agency has already confirmed that lactose-derived prebiotics are ‘safe’. For these reasons, they are often recommended for children, young and elderly individuals and even pregnant mothers. In many cases, medical practitioners recommended the consumption of probiotics and lactose-derived prebiotics together. It is believed that lactose-derived prebiotics are broken down by already existing intestinal probiotics and probiotic–prebiotic interactions reduce the risks of several gastrointestinal discomforts. In most of the cases negative side effects were not reported. The adverse effects of lactose-derived prebiotics are osmotic diarrhea, abdominal pain, and vomiting caused by an excess intake of prebiotics. The dose of prebiotic treatment is age- and case-dependent. Doses of galacto-oligosaccharide or lactulose or lactitol are generally adjusted to ensure 2–4 bowel movements per day. There are many biochemical mechanisms related to probiotic and lactose-derived prebiotic interactions that are still unclear, and their successful validation has not yet been proven. Therefore, more standardized and verifiable clinical studies are needed to demonstrate the safety, efficacy and limitations of putative lactose-based prebiotics. 

The lactose concentration in whey is 4–4.5% (*w*/*v*), which is responsible for its high biological oxygen demand and chemical oxygen demand values. Instead of the direct disposal of whey into the aquatic system, there is a great opportunity to produce different types of lactose-derived prebiotics, such as galacto-oligosaccharide, lactulose, lactosucrose, tagatose, lactitol, lactobiono- and glucono-δ-lactone from whey through different chemical and biochemical reactions as well as microbial fermentation processes. This may implement ‘zero waste disposal’ of dairy effluent and ‘waste valorization’. Low cost feedstock may bring down the operating cost and reduce the pay-back time of the technology. 

It is hoped that this review will received great attention from medical practitioners, and food and nutrition research communities. Moreover, the present review will open a new arena in the cutting-age research area of biotechnology and will serve as a ready reference for future research communities and scientists.

## Figures and Tables

**Table 1:** Criteria and specific signs of a healthy gastrointestinal system (self-developed, information was collected from Bischoff, 2011 [16]).

Criteria for a Healthy Gastrointestinal System	Specific Signs of Gastrointestinal Health
Effective digestion of food and absorption of nutrients	Effective absorption of food derivatives, water and minerals Regular bowel movements and normal transit time with usual stool consistency No abdominal pain and indigestion
Non-appearance of gastrointestinal illness	Absence of gastroesophageal reflux disease (heartburn or acid indigestion), diarrhea, nausea, vomiting, bloating and constipation Absence of lactose-, cereal food- and protein-intolerance No colorectal or other gastrointestinal tumor and cancer
Stable and substantial growth of intestinal flora	No pathogenic overgrowth in gastrointestinal tract and hepatic encephalopathy No bowel disorders (antibiotic-associated diarrhea, traveler’s diarrhea, acute watery diarrhea and *Clostridium difficile* associated relapsing diarrhea) and inflammatory bowel diseases (Crohn’s disease and ulcerative colitis)
Effective immune status	Effective gastrointestinal barrier function, normal mucus secretion and absence of enhanced bacterial translocation Normal levels and activities of immunoglobulin A and immune cells Improve immune tolerance and no mucosal hypersensitivity or allergy
Overall comfort	Normal quality of life and its improvement Balanced serotonin secretion, normal function of the enteric nervous system and positive feeling in gut
